# The use of a medical application improves the diagnosis of acute kidney injury: A pre-post study

**DOI:** 10.3389/fmed.2022.817387

**Published:** 2022-08-16

**Authors:** Andrea Gaspar, Maria F. Iturricha-Cáceres, Etienne Macedo, Ravindra L. Mehta, Rolando Claure-Del Granado

**Affiliations:** ^1^MedStar Franklin Square Medical Center, Baltimore, MD, United States; ^2^Facultad de Medicina, Universidad Privada del Valle, Tiquipaya, Bolivia; ^3^Division of Nephrology-Hypertension, University of California, San Diego, San Diego, CA, United States; ^4^Facultad de Medicina, Universidad Mayor de San Simón, Cochabamba, Bolivia; ^5^Hospital Obrero No 2 - CNS, Cochabamba, Bolivia

**Keywords:** acute kidney injury, medical application, serum creatinine, mobile health, smart-phones

## Abstract

The use of mobile devices by healthcare providers has transformed many aspects of clinical practice. Mobile devices and medical applications provide many benefits, perhaps most significantly increased access to point-of-care (POC) tools, which has been shown to support better clinical decision making and improved patient outcomes. In LMICs, where computer-based technology is limited, the use of mobile technology has the potential to immensely increase access to point of care tools. In this study, we conducted an interventional, pre-post study to determine whether the use of a medical application could help healthcare providers accurately recognize and diagnose AKI. After preparing 20 clinical vignettes based on AKI cases from our center Global Snapshot study report, we asked 50 last year medical students to identify the presence and stage of AKI first without and then with the use of the IRA SLANH App (IRA SLANH app, Island of the Moon^®^ V.1, 2014; Cochabamba-Bolivia), which was designed specifically for this study. Before the IRA SLANH app was introduced, the mean number of correctly identified cases of AKI was 14.7 ± 4.7 with a minimum of 3 and a maximum of 20. The stage of AKI was correctly identified in only 6.7 ± 4.4 of the cases. After the app was introduced, the number of correctly identified and staged cases of AKI was 20. Medical applications are useful point-of-care tools in the practice of evidence-based medicine. Their use has the potential to play a very important role in early identification and classification of AKI, particularly in LMICs potentially allowing for earlier intervention with preventive and treatment strategies to reverse kidney injury and improve recovery.

## Background

Acute kidney injury (AKI) is a common problem worldwide, affecting over 13 million people annually and causing 1.7 million deaths (1–3). AKI is caused by a multitude of etiologies (i.e., nephrotoxic medications, antibiotics, contrast, dehydration, sepsis, heart failure) and can occur in the hospital or in the community. Hospital-acquired AKI is often multifactorial and occurs in patients that have multiple comorbidities that augment susceptibility to AKI (4–6). In contrast, community-acquired AKI usually occurs after just one inciting event, such as a diarrhea or an infectious illness, and it affects those with fewer comorbidities (6). Every episode of AKI increases the risk for progression to chronic kidney disease, end-stage kidney disease, and death - all of which can lead to diminished quality of life for patients, higher healthcare costs, and strain on healthcare systems. More severe AKI translates into a greater risk (3, 4). If identified and managed in a timely fashion, however, AKI is treatable and reversible, preventing its numerous burdensome sequelae.

AKI is particularly problematic in low-and-middle-income countries (LMICs), where rates are significantly higher compared to high-income countries (HICs) and healthcare systems are chronically resource-constrained. Nearly 85% of the world's cases of AKI occur in low-and-middle-income countries (LMICs). Despite the fact that most of these cases are caused by a single preventable insult such as a diarrheal illness or dehydration, associated morbidity and mortality is higher (1, 3, 7, 8). This is often a result of the inability to rapidly diagnose and treat AKI due to inadequate laboratory facilities, poor knowledge about risk factors for AKI and its consequences, as well as a dearth of supplies to aggressively manage AKI. Moreover, AKI more often occurs in younger patients with fewer co-morbidities, individuals whose disability from illness potentially renders a significant burden on society.

Because AKI is treatable and reversible if intervened upon early, it is important to find innovative ways to mitigate the burden of unrecognized AKI in LMICs. One potential way to do this is through using mobile technology (mHealth) to create point-of-care tools that allow healthcare providers to correctly recognize, stage, and treat AKI (3). Because mHealth bypasses the need for computers and Internet – resources which are often limited or non-existent in LMIC clinical settings - it is a promising tool to improve access to point-of-care testing and decision making (9).

In this study, we employed mHealth to help healthcare providers recognize and classify AKI. We created a Smartphone-based application (IRA SLANH app, Island of the Moon^®^ V.1, 2014; Cochabamba-Bolivia) to assist with AKI diagnosis, and we hypothesize that its use would improve both recognition and classification of AKI.

## Methods

The primary objective of this study was to evaluate the utility of a Smartphone-based mobile application in helping healthcare providers accurately diagnose and stage AKI. In order to do this, we conducted an interventional pre-post study in which last-year medical students from Cochabamba, Bolivia, were asked to identify the presence and stage of AKI both with and without the use of the IRA-SLANH application. The primary outcome was the percentage of medical students correctly identifying and staging AKI before and after use of the application.

Hospital Obrero No 2 Caja Nacional de Salud is a government-run hospital serving essential employees located in Cochabamba, Bolivia. Using the hospital's Global Snapshot Study Report, we identified twenty confirmed cases of AKI by comparing a baseline SCr to another SCr measurement within seven days of hospital admission. AKI was defined using the KDIGO guidelines of a >0.3 rise in SCr within 48 h or an increase in SCr to > 1.5 times baseline occurring within the last seven days (10). Prior to initiation of the study, approval by the local ethics committee (Hospital Obrero No 2 - C.N.S, ethics committee) and written consent to participate from all participants was obtained. All procedures were in accordance with the Declaration of Helsinki.

**Figure 1:**
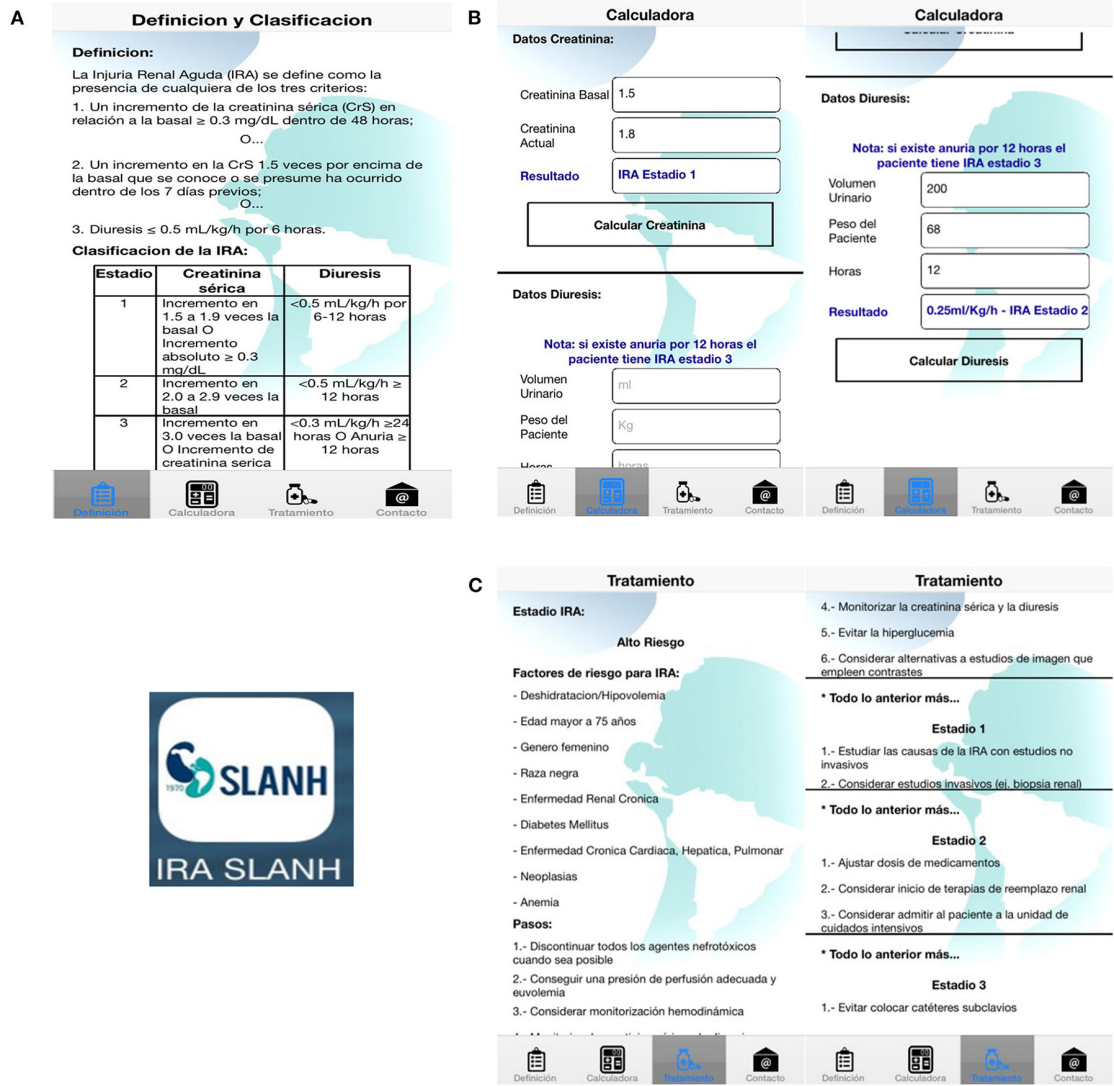
Different tabs of the IRA SLANH application. **(A)** The first tab reviews the KDIGO criteria for the definition and stages of AKI (11). **(B)** The second tab calculates the presence and stage of AKI according to these guidelines; it uses either the patient's urine output or a comparison of their baseline sCr to current sCr. **(C)** The third tab provides recommendations for clinical management according both to the level of the AKI, also based on the current KDIGO guidelines (11).

**Figure 2:**
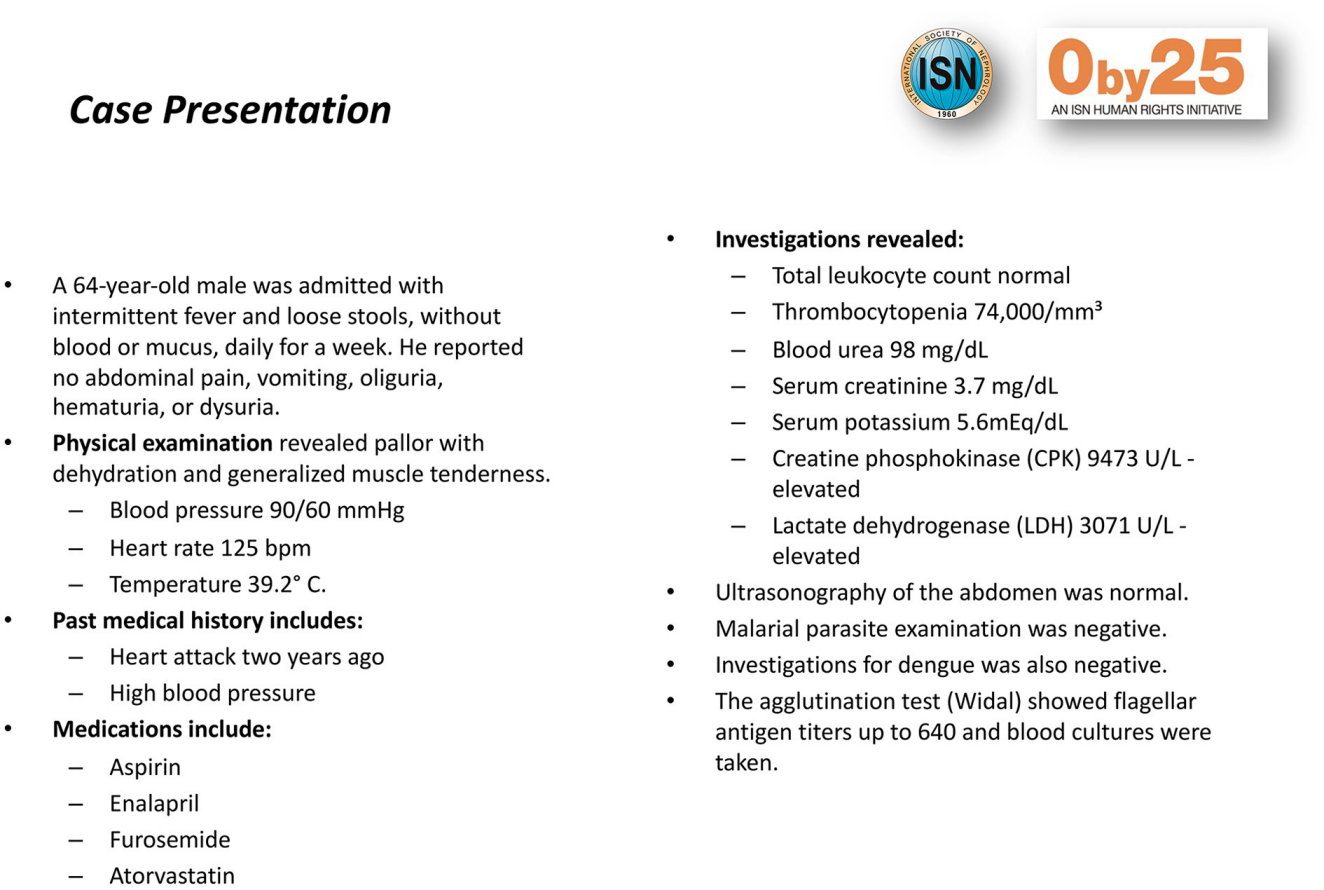
Clinical vignettes. Example of one of the clinical vignettes prepared for the study.

Study participants were selected *via* convenience sampling. Any last-year medical student attending Cochabamba's medical school was eligible. All available students participated for a total of 50 students. Clinical vignettes about each of the pre-identified 20 cases of AKI were prepared (Figure 1). Patient demographics and risk factors for AKI were included in the vignettes (Table 1 shows some characteristics of patients with AKI). The medical students were asked to read the vignette and identify both the presence and stage of AKI in each case. After 72 h, they were then asked to download the IRA-SLANH application. The IRA-SLANH application is unique in that it is one of the few smart-phone based applications that assist with identifying the presence and stage of AKI as well as recommended treatment. It is free and available to all healthcare providers who have a Smartphone. All information presented in the application is based on the KDIGO criteria and guidelines of identifying and managing AKI (10). Figure 2 show the functioning of the application in greater detail.

**Table 1:** Characteristics of acute kidney injury.

**Documented location**		
	**Percentage**	
Community-acquired AKI	(56.4%)	
Hospital-acquired AKI	(43.6%)	
**AKI Stages**		
AKI Stage 1	37.9%	
AKI Stage 2	19.2%	
AKI Stage 3	42.9%	
**Documented etiologies**		
	**Number (n)**	**Percentage (%)**
Dehydration	11.8	59%
Hypotension/shock	11.28	56.4%
Cardiac Disease	2.56	12.8%
Liver Disease	1.54	7.7%
Urinary obstruction	2.06	10.3%
Infection	2.56	12.8%
Nephrotoxic Agents	9.74	48.7%
Animal Venom	0.52	2.6%
Sepsis	10.76	53.8%

^*^More than one risk factor could be present in the same patient. Community-acquired AKI is usually more common in LMICs, and usually present in more advance stages (AKI stage 2–3 in 62.1%) due to delay recognition and diagnosis. The three most common causes of AKI were dehydration, hypotension, and nephrotoxins.

After downloading the application, the students were asked again to identify and stage AKI, and the number of correctly identified cases and percentage of students identifying all cases correctly were compared continuous variables were expressed as the mean ± SD, or median and interquartile range. Between-group comparisons of continuous variables were performed using the independent *t*-test or Mann-Whitney *U* test, after testing for normality using the Kolmogorov-Smirnov test. Dichotomous variables were compared using chi-square test or Fisher's exact test. All statistical analyses were two-tailed and performed using SPSS 22 software (SPSSFW, SPSS Inc., IBM, Armonk, NY, USA). The two-sided *P* < 0.05 was considered statistically significant.

## Results

The most common comorbidity among patients identified with AKI in this study was diabetes mellitus (28.2%). Mean creatinine was 0.9 with a range of 0.8–1.1 mg/dL. The most common etiologies of AKI included dehydration (59%), hypotension/shock (56.4%), and nephrotoxic agents (48.7%). Just over half (56.4%) of cases occurred in the community, and the remainder (43.6%) occurred in the hospital. Prior to the introduction of the IRA-SLANH application, 22% of medical students correctly identified the presence of AKI in all cases, and 0% identified its stage in all cases. The mean number of correctly identified AKI cases was 14.7 +/−4.7 (Table 2), and the mean number of correctly staged cases was 6.7 +/−4.4. With use of the application, 100% of students were able to correctly identify and stage AKI in all 20 cases.

**Table 2:** AKI recognition and classification pre and post *IRA-SLANH App*.

	**AKI recognition**		**AKI classification**	
	**Pre App**	**Post App**	**Pre App**	**Post App**
Mean ± SD of correct answers	14.7 ± 4.7^a^	20^a^	6.7 ± 4.4^b^	20^b^
Minimum number of correct answers	3	20	0	20
Maximum number of correct answers	20	20	16	20

Only 22% of students could correctly identified AKI in all 20 cases. 0% of students could correctly classified (stages 1, 2, or 3) all AKI cases.

^*^a and ^*^b p < 0.001. The use of a the IRA-SLANH App improved the recognition and classification of AKI in a low-resource setting.

## Discussion

The use of our app (IRA-SLANH) improved the ability of medical students to identify and stage AKI. The ease of access to information enabled them to quickly identify and stage AKI based on the KDIGO criteria. We showed that a simple intervention with a smartphone-based medical application can have a meaningful impact on correct diagnosis of AKI.

Several studies have demonstrated that point-of-care tools can help providers identify and appropriately manage AKI. They also have important impacts on patient care by improving time to treatment, increasing rates of renal function recovery, and decreasing mortality (12, 13). Most of these studies, however, have been conducted in high-income countries using electronic medical records (12, 13). Due to cost and high technical skills requirements, electronic medical records are not commonly used in LMICs (14). It is therefore important to find other platforms to create point-of-care tools for AKI diagnosis and treatment.

The use of mobile technology, including Smartphones, is increasing throughout the world (15). Because mobile technology bypasses the need for computers and the Internet, it is a promising and cost-effective vehicle to improve access to medical information, including point-of-care decision tools through medical applications (16, 17). Along with being widely accessible through Smartphones, medical applications are appealing because minimal training is required for their use and running costs are negligible. The IRA-SLANH application is an example of a medical application that is simple in nature but has potentially profound impacts on both short-term and long-term patient outcomes. The downstream effects of early AKI recognition in LMICs will help diminish the burden that chronic sequelae such as chronic kidney disease and end-stage renal disease have on patients, healthcare systems, and society (6, 11).

To our knowledge, this is the first LMIC-based study that examines the use of a point-of-care tool to help providers accurately diagnose AKI. It is also one of the first studies to examine Smartphone-based point-of-care decision-making tools in LMICs (17). Given the prevalence of AKI in LMICs, its disproportionate morbidity and mortality, and its often-insidious presentation, equipping healthcare providers with easily accessible, point-of-care tools to quickly and accurately diagnose and manage AKI is crucial. The results of our study are promising that medical applications can play an important role in the early identification, staging, and prompt initiation of AKI treatment.

The main strength of this study is that it is one of the first studies of its kind in many realms. It is novel in its evaluation of a Smartphone-based point-of-care tool of LMICs and its innovative approach to AKI diagnosis. It is one of the few - if not only - medical applications available that not only identifies AKI but also stages it and recommends guideline-based treatment. Additionally, the significant differences observed before and after use of the application lay a strong foundation for future studies to more extensively examine how point-of-care tools assist with diagnosis and treatment of AKI in LMIC settings and affect patient outcomes.

This study has several limitations. Although it was an interventional study, it was a non-randomized pre-post design. We did not assess knowledge of AKI prior to the study or randomize students based on other characteristics. The study size was also small. Although the study was limited to trainees whose knowledge and skills are not as refined as fully trained providers, they were in their last year of training and most likely had gained the majority of the knowledge needed to practice independently. In addition, the study did not investigate how improved recognition of AKI impacted management or clinical outcomes. Future studies are needed to investigate these important aspects, especially since AKI is a condition whose potentially grave consequences are easily reversible if recognized early.

## Conclusions

Smartphone-based medical applications have the potential to be incredibly useful point-of-care tools for helping LMIC providers apply evidence-based medicine when managing AKI. The significant improvement we saw in the ability of students to accurately diagnose AKI before and after the use of the IRA-SLANH application shows how a simple, low-cost intervention can potentially have a hugely positive impact. Although our study was small, our findings lay a strong foundation for future studies to investigate the feasibility and impact of point-of-care tools to assist with diagnosis and management of AKI in LMIC settings.

## Data availability statement

The raw data supporting the conclusions of this article will be made available by the authors, without undue reservation.

## Ethics statement

The studies involving human participants were reviewed and approved by Jefatura de Enseñanza e Investigación Hospital Obrero No 2 - CNS. The patients/participants provided their written informed consent to participate in this study.

## Author contributions

RC-D: study concept and design, statistical analysis, obtained funding, and study supervision. MI-C, AG, and RC-D: data acquisition, analysis, or interpretation of data. AG, MI-C, RM, EM, and RC-D: drafting of the manuscript. AG, RM, EM, and RC-D: critical revision of the manuscript for important intellectual content. MI-C and RC-D: had full access to all of the data in the study and take responsibility for the integrity of the data, the accuracy of the data analysis, the honest, accurate, and transparent reporting of the study. All authors contributed important intellectual content during manuscript drafting or revision and accepts accountability for the overall work by ensuring that questions pertaining to the accuracy or integrity of any portion of the work are appropriately investigated and resolved. All authors read and approved the final manuscript.

## Funding

The clinical study reported in this publication was supported by a Sister Renal Center Program grant from the International Society of Nephrology (ISN) who provided research support for the implementation of the 0by25 pilot study at our Institution. The IRA SLANH^®^ app development was supported by a grant provided by the Acute Kidney Injury Committee of the Latin American Society of Nephrology and Hypertension (SLANH), this grant cover the cost of the design and the development of the application by the apps developer company (Island of the Moon, Cochabamba-Bolivia).

## Conflict of interest

The authors declare that the research was conducted in the absence of any commercial or financial relationships that could be construed as a potential conflict of interest.

## Publisher's note

All claims expressed in this article are solely those of the authors and do not necessarily represent those of their affiliated organizations, or those of the publisher, the editors and the reviewers. Any product that may be evaluated in this article, or claim that may be made by its manufacturer, is not guaranteed or endorsed by the publisher.

## Abbreviations

POC: point-of-care
sCr: serum creatinine
AKI: Acute kidney injury
CKD: Chronic Kidney Disease
ESKD: End Stage Kidney Disease
HICs: high-income countries
LMICs: low- and middle-income countries
RRT: renal replacement therapy
mHealth: Mobile health
KDIGO: Kidney Disease: Improving Global Outcomes.
